# Genetic factors and the risk of drug-resistant epilepsy in young children with epilepsy and neurodevelopment disability: A prospective study and updated meta-analysis

**DOI:** 10.1097/MD.0000000000025277

**Published:** 2021-03-26

**Authors:** Chien-Heng Lin, I-Ching Chou, Syuan-Yu Hong

**Affiliations:** a Division of Pediatrics Pulmonology, China Medical University, Children's Hospital, Taichung, Taiwan; b Department of Biomedical Imaging and Radiological Science, College of Medicine, China Medical University; c Division of Pediatrics Neurology, China Medical University, Children's Hospital; d Graduate Institute of Integrated Medicine, College of Chinese Medicine, China Medical University, Taichung, Taiwan.

**Keywords:** children, drug-resistant epilepsy, genetic, infant, neurodevelopmental disability

## Abstract

Drug-resistant epilepsy (DRE) affects 7% to 20% of children with epilepsy. Although some risk factors for DRE have been identified, the results have not been consistent. Moreover, data regarding the risk factors for epilepsy and its seizure outcome in the first 2 years of life are limited.

We analyzed data for children aged 0 to 2 years with epilepsy and neurodevelopmental disability from January, 2013, through December, 2017. These patients were followed up to compare the risk of DRE in patients with genetic defect (genetic group) with that without genetic defect (nongenetic group). Additionally, we conducted a meta-analysis to identify the pooled prevalence of genetic factors in children with DRE.

A total of 96 patients were enrolled. A total of 68 patients were enrolled in the nongenetic group, whereas 28 patients were enrolled in the genetic group. The overall DRE risk in the genetic group was 6.5 times (95% confidence interval [CI], 2.15–19.6; *p* = 0.03) higher than that in the nongenetic group. Separately, a total of 1308 DRE patients were participated in the meta-analysis. The pooled prevalence of these patients with genetic factors was 22.8% (95% CI 17.4–29.3).

The genetic defect plays a crucial role in the development of DRE in younger children with epilepsy and neurodevelopmental disability. The results can serve as a reference for further studies of epilepsy panel design and may also assist in the development of improved treatments and prevention strategies for DRE.

## 1 Introduction

Data regarding epilepsy and its outcome in children aged < 2 years are limited.^[1]^ With proper and adequate treatment, childhood epilepsy can achieve remission in 60% to 70% of the cases, with nearly 50% being able to discontinue antiepileptic drug (AED) use.^[2–4]^ Unlike epilepsy occurring in later childhood or adulthood, seizures occurring in developing brain have greater influence in the alteration of neurogenesis, synaptogenesis, excitatory/inhibitory balance and network connectivity, which enable the early*-*life epilepsies to present more clinically complex. Additionally, multifarious genetic etiology diversifies the clinical picture of epilepsy during this age, which makes it etiologically heterogenous.^[3–5]^ Therefore, the prediction of seizure outcomes during infancy is relatively difficult compared with that beyond infancy. Given the advancement of molecular bioassay technology, an increasing number of genetic etiologies underlying infantile epilepsy have been found.

A meta-analysis of 35 studies on patients with drug-resistant epilepsy (DRE) revealed the pooled prevalence and pooled incidence of DRE across all age groups of epilepsy to be 30% and 15%, respectively.^[5]^ Among adults, the risk factors for DRE included abnormal electroencephalograms (in terms of both epileptiform and slow wave discharges), symptomatic etiologies, febrile seizures, status epilepticus, the presence of developmental delay, and multiple seizure types.^[6,7]^ However, the risk of DRE in infancy and younger children remain uncertain thus far given the heterogeneous etiologies as well as misdiagnoses.^[5]^

Identifying the causes underlying epilepsy in the first 2 years of life can be challenging for pediatric neurologists. Determining the etiology of epilepsy can aid in predicting the neurodevelopmental outcomes and seizure control in children in addition to the choice of AEDs. Herein, we report a study exploring the incidence of DRE in 0–2-year-old infants and children under a new perspective. To determine DRE incidence, the factors leading to young children epilepsy are classified into genetic and nongenetic factors, with the patients in both groups demonstrating neurodevelopmental disability (NDD). Additionally, in order to clarify the role of genetic factor playing in childhood DRE, we searched for studies which explored etiology associated with childhood DRE and a meta-analysis was conducted to serve as a counter-directional comparison for our present study.

## 2 Patients and methods

### 2.1 Patient population

A comprehensive medical review of patients aged <2 years diagnosed as having epilepsy and NDD between January 1, 2013 and December 31, 2017 in the China Medical University Children's Hospital was conducted. These children (and their parents) were in contact by our case managers and underwent regular follow-up at our Pediatrics Neurology Clinic until December 31, 2018; recruitment of the children to this study was conducted after approval of the study was obtained from our institutional ethics committee (CMUH108-REC1-023). The patients (if equal to or more than 7 years old) and their parents provided their assent and written informed consent, respectively, before their enrolment. The definition of DRE was referred from the Task Force of the International League Against Epilepsy (ILAE): “The failure of adequate trials of two tolerated, appropriately chosen, and administered AEDs (whether as monotherapy or in combination) to achieve seizure freedom.^[8]^” Moreover, adequate seizure control was defined as the patient remaining seizure-free for either at least 2 months or for 2 times the length of the usual pretreatment interictal interval, whichever was longer. Poor or partial seizure control in children was defined as the occurrence of more than 1 seizure per month over a minimum of 6 months, as indicated by Chawla et al.^[9,10]^ The “Rule of Three” proposed by 2012 ILAE task force was used as the operational definition for seizure freedom: that is, a patient should only be regarded as seizure-free subsequent to an intervention when a seizure-free period that is 3 times longer than the longest interseizure interval over the previous year before the intervention has elapsed.^[11]^ All patients were followed up for at least 12 months, which included at least 3 visits to the Pediatrics Neurology Clinic, whereas patients who had intractable seizure due to untreated or incomplete treatment of an underlying disease, demonstrated poor drug compliance, died or were lost to follow-up, or received nonepileptic drug treatment were excluded. The enrolled patients (n = 96) underwent a series of laboratory tests; they also maintained records and individual seizure diaries, which contained details about sex, preterm or full-term pregnancy, age at seizure onset, underlying cause of epilepsy, age at the time when AED therapy was initiated and the number of AEDs taken, seizure-free status, family history of any seizure disorder, electroencephalograms pattern at the time of the first diagnosis of epilepsy, comorbidities, and neurodevelopmental outcomes.

**Figure 1:**
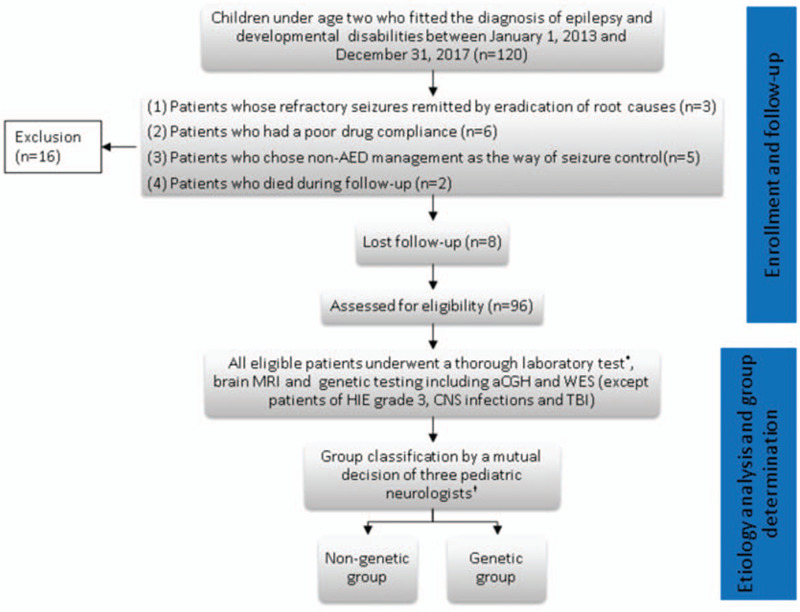
Study flowchart. aCGH = array comparative genomic hybridization, MRI = magnetic resonance imaging, WES = whole-exome sequencing. ^∗^The investigation was comprehensive and included analyses of and the performance of, respectively, the following factors and procedures: arterial blood gas, pH, lactate, and pyruvate levels; complete serum biochemistry testing; analysis of cerebrospinal fluid; and toxicology screening. ^†^Causes of epilepsy were determined by 3 independent pediatric neurologists, in case of inconsistent conclusions, patients were classified as unknown in the nongenetic group.

According to our study protocal (Fig. 1), all eligible patients underwent series of neuroimage and laboratory tests to search for the etiology. As a result, their causes of epilepsy were categorized into genetic and nongenetic group by 3 independent pediatric neurologists. The patients of the genetic group were those in whom epilepsy was because of pathogenic single-gene mutations or defined structural chromosomal aberrations, such as microdeletion or microduplication.

### 2.2 Search strategy and selection criteria for meta-analysis to studies regarding genetic factors among childhood DRE

We complied with the guidelines of the Preferred Reporting Items for Systematic Reviews and Meta-Analyses to conduct and report this meta-analysis. Electronic databases—namely Embase, MEDLINE, Web of Science, and Google Scholar—were searched using the terms (“intractable” OR “medically refractory” OR “pharmacoresistant” OR “drug-resistant”) AND (“Epilepsy” OR “seizure disorders”) AND (“Pediatric” OR “Infant” OR “Infancy” OR “Neonatal” OR “Childhood”). The databases were searched for studies published after 2014. Studies were included in accordance with the following criteria:

(1) Retrospective or prospective studies with regard to etiology of childhood DRE approached with sound methods and full text published in English,
(2) studies with diagnosis of DRE based on the definition referred from the Task Force of the International League Against Epilepsy (ILAE) in 2009.^[8]^

**Figure 2:**
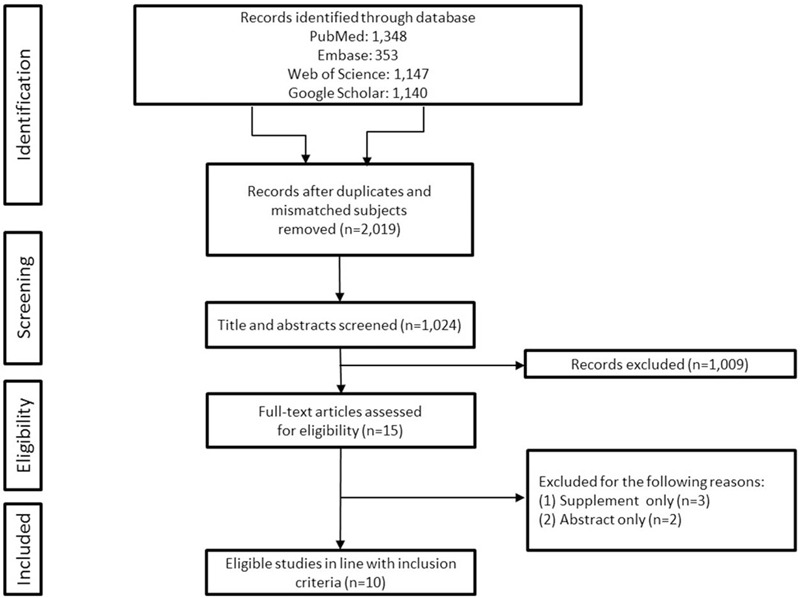
Flow diagram of the search process and search results.

Unpublished data, supplement data, conference abstracts, reviews, and editorials were also excluded (Fig. 2).

### 2.3 Data extraction and study quality assessment

Two reviewers (SYH, ICC) independently screened the titles and abstracts of studies to identify trials that met the inclusion criteria. When disagreements occurred between reviewers, the full text of the paper was retrieved, and the disagreements were discussed until a consensus was reached. The same 2 reviewers respectively extracted the data using a standard data extraction protocol. Information extracted included the study characteristics (e.g., title, the first author, publication year, study site, survey time, sample size, epilepsy subtype, age, and gender, diagnostic tools, and data on the prevalence of genetic causes of DRE

### 2.4 Statistical analysis

In the prospective study, Chi-squared tests were used to analyze the differences in categorical variables between the groups, while the DRE incidence density rates were also calculated for both groups. In addition, the odds ratios (ORs) and 95% confidence intervals (CIs) of DRE in the genetic group versus the nongenetic group were estimated through the application of a logistic regression model. The PASW Statistics software (version 18.0; SPSS Inc., Chicago, IL, United States) was used to perform all the statistical analyses, with a 2-tailed *P* value of <.05 being regarded as statistically significant.

In the meta-analysis, we used comprehensive meta-analysis statistical software to analyze the data. Due to the heterogeneity in sampling methods, assessment instruments and sample size across studies, random-effects model was used to estimate the pooled prevalence of genetic causes among DRE. The standard chi-squared test and *I*^2^ statistics were used to evaluate the consistency of the research results. Evidence of publication bias was assessed with funnel plots

## 3 Results

### 3.1 Data analysis

**Figure 3:**
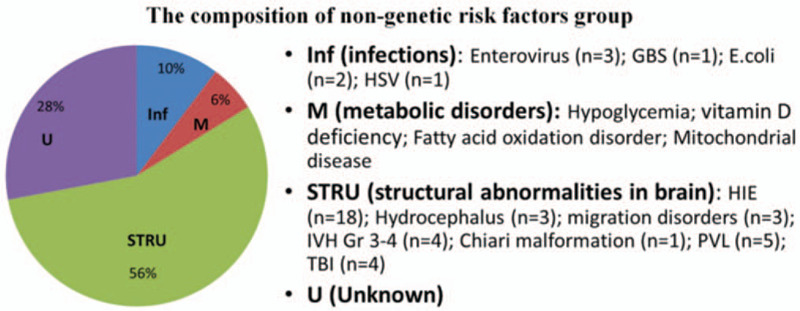
Composition of nongenetic risk factors group. E.coli = Escherichia coli, GBS = group-B streptococcus, HIE = hypoxic–ischemic encephalopathy, HSV = herpes simplex virus, Inf = infections, IVH = intraventricular hemorrhage, M = metabolic disorders, PVL = periventricular leukomalacia, STRU = structural abnormalities in brain, TBI = traumatic brain injury, U = unknown.

**Figure 4:**
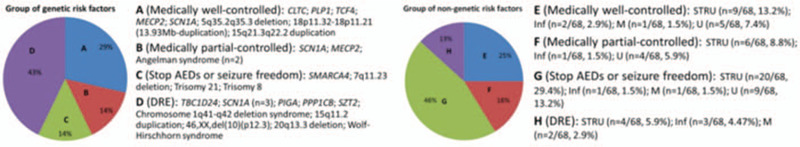
Seizure control status and their proportions in genetic and nongenetic groups. Inf = infections, M = metabolic disorders, STRU = structural abnormalities in brain, U = unknown.

Between January 1, 2013 and December 31, 2017, 96 children aged <2 year with epilepsy and NDD were enrolled in this study (Fig. 1). There are 68 children in nongenetic group and 28 children in genetic group. Table 1 presents their demographic characteristics: mean (standard deviation [SD]) age at epilepsy diagnosis and follow-up duration were respectively 7.54 (5.25) months and 2.64 (0.73) years in the genetic group and 4.96 (3.99) months and 2.93 (1.04) years in the nongenetic group. The nongenetic group was divided into 4 subgroups (Fig. 3): abnormal brain structure (n = 38, 55.8%), infections (n = 7, 10.2%), metabolic disorders (n = 4, 5.8%), and unknown (n = 19, 27.9%). The genetic group was divided into 2 subgroups, there are 17 children belong to single-gene mutations, and 11 chromosome abnormalities. Moreover, DRE incidence was 42.8% and 13.2% in the genetic and nongenetic groups, respectively (Fig. 4).

**Table 1:** Demographic and clinical characteristics of children with epilepsy and neurodevelopmental disabilities in the genetic and non-genetic groups.

	Group		
	Genetic, n = 28 (%)	Non-genetic, n = 68 (%)	*P*
Gender, Male	12 (42.9)	40 (59.7)	.13
Preterm (%)	4 (14.2)	13 (19.1)	.57
FHx of seizure disorders (%)	2 (7.1)	2 (2.9)	.34
Age of epilepsy Dx (mo) (SD)^∗^	7.54 (5.25)	4.96 (3.99)	.01
Abnormalities other than the CNS (%)	12 (42.8)	12 (17.6)	.10
Facial and outward appearance	8 (28.5)	4 (5.8)	–
Cardiovascular	5 (17.8)	7 (9.8)	–
Genitourinary system	4 (14.2)	1 (1.4)	–
Positive MRI findings (%)	8 (28.5)	44 (64.7)	.003
1st interictal EEG patterns (%)			.38
Negative	9 (32.1)	11 (16.1)	–
Epileptogenic discharges	13 (46.4)	43 (63.2)	–
Slow	3 (10.7)	13 (19.1)	–
Hypsarrhythmia and/or burst suppression	3 (10.7)	1 (1.4)	–
F/U year (SD)^∗^	2.64 (0.73)	2.93 (1.04)	.19
Prenatal, perinatal or postnatal risk factors for epilepsy			<.001
Genetic	28 (100)	0	–
Abnormal brain structure	0	38 (55.8)	–
Infections	0	7 (10.2)	–
Metabolic disorders	0	4 (5.8)	–
Unknown	0	19 (27.9)	–

AEDs = Anti-epileptic drugs, CNS = central nervous system, DRE = drug-resistant epilepsy, Dx = diagnosis, F/U = follow-up, FHx = family history, mo = month, MRI = magnetic resonance imaging.

∗ *t* test.

### 3.2 Difference in DRE incidence between genetic and nongenetic groups

Table 2 compares DRE relative risks and incidence rates for the nongenetic and genetic patient groups. The overall risk of DRE for the genetic group was greater than that for the nongenetic group (adjusted OR, 6.50; 95% CI, 2.15–19.6; *P* = .03). Furthermore, DRE risk in female patients in the genetic group was higher than that in those in the nongenetic group (adjusted OR, 8.88; 95% CI, 1.38–57.1; *P* = .03). In addition, full-term genetic group had a 7.1 times (95% CI, 2.02–24.9; *P* = 0.001) higher DRE risk than did full-term nongenetic group. Excluding those with unknown causes of epilepsy in the nongenetic group, the DRE risk in the genetic group was 4.3 times (95% CI, 1.18–9.43, *P* = .03) higher than the nongenetic group.

**Table 2:** Incidence rates and relative risks of drug-resistant epilepsy (DRE) for the infants with genetic-risk factor group and infants with non-genetic risk factor group and those stratified by sex and term and preterm neonates by using a logistic regression model.

	DRE			
Group	Event (No.)	IR (%)	OR (95% CI)	Adj. OR (95% CI)
Non-Genetic^†^ (n = 68)	9	13.2	Reference^†^	Reference^†^
Non-Genetic^‡^ (n = 49)			Reference^‡^	Reference^‡^
Sex				
M (n = 40)	6	8.8	Reference^†^	Reference^†^
F (n = 28)	3	4.4	Reference^†^	Reference^†^
Gestation				
Full term (n = 55)	7	10.3	Reference^†^	Reference^†^
Preterm (n = 13)	2	2.9	Reference^†^	Reference^†^
Genetic (n = 28)	12	42.8	4.91 (1.76, 13.7)^∗^	6.50 (2.15,19.6)^∗^
			3.33 (1.18, 9.43)^∗^	4.38 (1.41,13.6)^∗^
Sex				
M (n = 12)	6	21.4	5.66 (1.36, 23.5)^∗^	5.66 (1.35, 23.6)^∗^
F (n = 16)	6	21.4	7.50 (1.29, 43.6)^∗^	8.88 (1.38,57.1)^∗^
Gestation				
Full term (n = 24)	10	35.7	4.89 (1.57,15.2)^∗∗^	7.09 (2.02,24.9)^∗∗^
Preterm (n = 4)	2	7.1	5.50 (0.46,65.1)	5.48 (0.46,65.0)

Adj OR = *adjusted odds ratios*, CI = Confidence interval, DRE = drug-resistant epilepsy, F = female, IR = Incidence rate, M = male, Model adjusted by gestational age, sex, days of hospitalization.

∗ *P* < .05.

∗∗ *P* < .01.

† Whole non-genetic group (n = 68) and serves as corresponding comparative references for those of genetic group.

‡ Non-Genetic group but exclude those with unknown causes (n = 49) and serves as corresponding comparative references for those of genetic group.

### 3.3 Systematic review and updated meta-analyses

**Figure 5:**
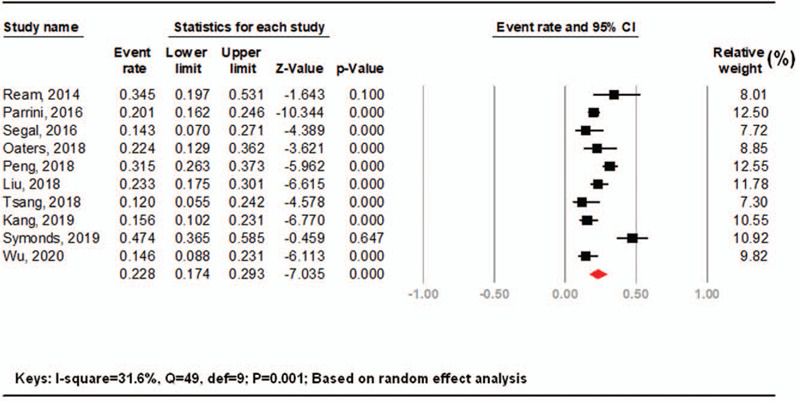
The forest plot of the prevalence of genetic causes of DRE in children.

After a rigorous screening, 10 relevant published studies for the genetic characteristics in DRE were included (6 with epilepsy next generation sequencing (NGS) panel; 1 with only whole exome sequencing (WES); 3 with WES plus array-based comparative genomic hybridization or NGS panel). Study algorithm is provided in (Fig. 2). Five studies were based from western countries (USA, Denmark, Italy and UK) and the other 5 were from Asia (China, Taiwan, south Korea, Hong Kong). Most participants of those studies were children (age <18 years), and since the molecular diagnostic tool (WES, NGS panel) become blooming over the past 6 years, the first included study was published in 2014. A total of 1308 DRE patients were participated in the meta-analysis and revealed that the pooled prevalence of in DRE patients of genetic factors was 22.8% (95% CI 17.4–29.3) (Fig. 5). The result showed a significant heterogeneity across all studies (*I*^2^ = 81.6%; *Q* = 49, df = 9, *P* < .001). The pooled prevalence was based on the random effect model due to the observed heterogeneity across the studies.

### 3.4 Genetic characteristics in drug-resistant epilepsy of published studies

Table 3 provides a summary of previous studies about genetic characteristics in DRE.^[12–21]^ Regardless of varied molecular diagnostic tool used between studies, some gene mutations were found to appear repeatedly, such as *SCN1A* (5.6%, n = 74/1308), followed by *SCN8A* (1.37%, n = 18/1308), *TSC2* (1.22%, n = 16/1308), *SCN2A* (1.07%, n = 14/1308) and *KCNQ2* (0.99%, n = 13/1308)

**Table 3:** Genetic characteristics in drug-resistant epilepsy of published research (2014–2020).

Study names, year	Research areas	Research objects with DRE, (n)	Research tools	The detected genetic or cytogenetics abnormalities (n)
Ream, 2014^[12]^	USA	Children (25)	karyotype, aCGH, single gene sequencing, Epilepsy NGS panels (38/40/53 genes)and WES	*PCDH19*(1), *SCN1A*(3), *SPTAN*(1), *SLC2A1* (1), *CDKL5* (1), *SLC9A6* (1), *EFHC1* (1), 69, XXX{28}/46, XX{22} (1), arr 2p25.3p25.1(2,772–10,840,014)x3 dn (1), 6q27(165,143,532–170,824,447)x1 dn (1)
Segal, 2016^[13]^	USA	Children (49)	Epilepsy NGS panels	*SCN1A* (3), *PCDH19* (2), *DLG3* (1), *MECP2* (1)
Parrini, 2016^[14]^	Italy	Children (349)	Epilepsy NGS panels (30 genes/ 95 genes)	*SCN2A*(9), *SCN1A*(8)*, KCNQ2*(6)*, STXBP1*(6)*, SCN8A*(5)*, CDKL5*(4), *MECP2*(4) and others^†^
Tsang, 2018^[15]^	Hong Kong	Children (50)	aCGH plus WES	*SCN8A* (1), *SCN1A*(1), *MECP2* (1), *CDKL5* (1), *DEPDC5* (1), *CHD2* (1)
Peng, 2018^[16]^	China	Children (273)	WES, MES, Epilepsy NGS panels (540 genes)	*SCN1A* (21), *SCN2A* (1), *SCN8A* (5), *GABRG2* (1), *KCNQ2* (3), *DOLK* (1), *KCNT1*(2), *TSC1*(4), *TSC2* (7), *PNPO*(1), *PCDH19*(3), *TRPM6*(1), *DNM1*(1), *SLC35A2*(1), *ALDH7A1* (1), *GNAO1*(1), *HCN1*(2), *KCNMA1*(4), *SLC6A1* (1), *SPTAN1*(1)
Liu, 2018^[17]^	China	Children <14 years (172)	Epilepsy NGS panels (153 genes)	*SCN1A* (16), *TSC2* (5), *STXBP1* (2), *SCN8A* (2), *TSC1*(1), *MECP2* (1), *CHD2* (1), *PCDH19* (1), *GABRA1* (1), *GABRB3* (1), *SLC2A1* (1), *SLC9A6* (1), *IQSEC2* (1), *KCNQ2* (1), *SCN2A* (1), *CACNA1A* (1), *KCNT1* (1), *SYNGAP1* (1), *ATP1A2* (1), *CDKL5* (1), *ADSL* (1), *VRK2* (1)
Oates, 2018^[18]^	Denmark	Children (96)	Epilepsy NGS panels (46/76/85/102 genes)	*SCN8A* (4), *SCN2A* (3), *SCN1A* (2), *KCNQ2* (2), *HNRNPU* (1), *GRIN2A* (1), *SYNGAP1* (1), *STXBP1* (1), *STX1B* (1), *CDKL5* (1), *CHRNA4* (1), *PCDH19* (1), *PIGT* (1).
Kang, 2019^[19]^	South Korea	Adults (122)	WES	*GABRG2*(2), *KCNT1* (1), *SCN1A* (3), *SCN9A* (1), *DEPDC5* (1), *TSC1*(2), *TSC2* (4), *ADGRV1*(1), *CNTNAP2*(2), *PRICKLE1*(1), *RELN*(3)
Symonds, 2019^[20]^	UK	Children under 36 months (76)	Epilepsy NGS panels (104 genes) and MLPA for relevant genes	*SCN1A* (12), *CDKL5* (4), *PCDH19* (4) and other 56 genes^†^
Wu, 2020^[21]^	Taiwan	Children (96)	Epilepsy NGS panels (24/122 genes)	*SCN1A*(5), *TBC1D24* (1), *KCNT1*(1), *KCNQ2*(1), *GRIN2A*(1), *ARX*(1), *ADSL*(1), *CHD2*(1), *SCN8A*(1)

^∗^aCGH = array comparative genomic hybridization, MLPA = multiplex ligation-dependent probe amplification, NGS = next generation sequencing, WES = whole exome sequencing.

† The authors did not list all the mutated genes in the paper. ^‡^The genes shown in bold are common gene mutation amid studies.

## 4 Discussion

Childhood DRE risk is serious and can have catastrophic effects on neurodevelopment.^[22,23]^ The brain undergoes an extended period of growth and maturation during the first 2 years of life. Thus, intractable seizures are refractory during this critical time, particularly in the early infancy, can negatively affect the cognitive and motor development, consider by affecting the children's cognitive, behavioral, and psychiatric function.^[24–27]^ Here, we investigated the risk of DRE in infants and younger children in a tertiary hospital over 5 years and noted a relatively higher risk of DRE combined with NDD caused by genetic factors (6.5 times (95% CI, 2.15–19.6). By contrast, the risk of DRE caused by nongenetic factors was relatively low, even in those with severe NDD.

Our results also demonstrated heterogenous genetic causes of epilepsy which range from neonate to late infancy (29.1%, n = 28/96). Single-gene mutations and cytogenetic abnormalities accounted for genetic causes of specific epilepsy phenotypes or selected recognizable syndromes with a high prevalence of seizures. Seizure control in this group was relatively difficult, and therefore, this group demonstrated a high DRE incidence rate (42.8%, n = 12/28), and the high risk of NDD.

To our knowledge, we carried out the first meta-analysis with regards to genetic causes of DRE. From the meta-analysis, we found high incidences of gene related DRE in children and infancy (pooled prevalence 22.8%, 95% CI 17.4–29.3). The result is consistent with our study although with different methodology. It goes without saying that we could not encompass all epilepsy genes in a single research and then estimate the incidence of DRE, and besides, the detected gene mutations vary between studies. Even so, after combining the 2, bidirectional comparison as a result, we demonstrate that genetic factors play a crucial role in childhood and infantile epilepsy and otherwise provided a reliable evidence to make believe a high probability of DRE existing in patients with genetic factors during childhood and infancy

Additionally, we discovered some common genetic mutations among childhood DRE through our study (n = 96) and the meta-analysis (n = 1308). Namely, SCN1A, which presented in almost every study including ours, and the others were *PCDH19*, *SCN8A, SCN2A, MECP2, KCNQ2, CDKL5, TSC1 and TSC2* (Table 3). Even now that molecular diagnostic tools are cutting prices for competition, the cost factor is still a major concern for most clinician and patients of DRE who sought for an accurate diagnosis for treatment.^[28]^ Hopefully, this result could serve as an informative reference for future DRE panel design to make it cost effective and more efficient, especially in which WES or comprehensive epilepsy panels are not easily available or affordable.

Perinatal and prenatal insults are major risk factors for infantile epilepsy.^[29]^ The proposed pathophysiological mechanism is that hypoxia–ischemia that can have deleterious effects on the vulnerable regions of the developing brain lead to substantive injuries that could affect not only seizure threshold but also cognition.^[30]^ Studies have also explored the mechanisms underlying neuronal injury, which could be a cause of epilepsy; first, hypoxic–ischemic encephalopathy (HIE) initially affects various processes that potentially contribute to energy failure and loss of mitochondrial function, including brain edema, membrane depolarization, increased levels of neurotransmitter release and uptake inhibition, and increased levels of intracellular calcium (which can cause the initiation of further pathological cascades).^[31]^ Second, excitotoxic cellular injury occurring through excess activation of the 4 glutamate receptors (N-methyl-d-aspartate, alpha-amino-3-hydroxy-5-methyl-4-isoxazoleproprionic acid, kainate, and metabotropic glutamate receptors), which leads to several forms of cell death, is another possible seizure mechanism associated with HIE.^[32–34]^

Traumatic brain injury, whether accidental or inflicted, is another common cause of epilepsy development in infancy.^[35]^ Notably, traumatic brain injury often coexists with HIE, which worsens the condition of the already fragile brain through molecular injury mechanisms similar to those of HIE,^[36,37]^ including excitotoxicity mediated by neurotransmitters that results in glutamate, free-radical injury to cell membranes, mitochondrial dysfunction, electrolyte imbalance, inflammatory response, focal microvascular occlusion, secondary ischemia from vasospasm, apoptosis, and vascular injury. These mechanisms result, in turn, in neuronal cell death concomitant with cerebral edema and an elevated risk of epilepsy.^[38–40]^

Gene-related epilepsy involves various heterogenous seizure mechanisms that depend on the role of the genes. There are over 970 genes associated with epilepsy, and the number is increasing year by year.^[41,42]^ Although the seizure mechanisms are complex and diverse between the causative genes, we could roughly categorize them into ion and nonion channel genes. Given the role played by epigenetics in neuronal function from the time of embryogenesis and early brain development, as well as in tissue-specific gene expression, epigenetic regulation also contributes to neurodevelopment through gene–environment interaction influencing epilepsy occurrence. The same principle is likewise applicable to localized multiple loci in which susceptibility genes to epilepsy are harbored and can explain epilepsy cases with cytogenetic abnormalities.^[43]^

Drug resistance mechanisms in epilepsy remain unclear. Margineanu DG et al proposed 2 current hypotheses that underscore the roles played by changes in the targets of medications that render them drug-insensitive and by the elevated actions of blood-brain barrier multidrug transporter proteins. However, the hypotheses in question do not seem to adequately account for the complicated nature of the brain alterations that occur in DRE.^[44]^ The current consensus on is that DRE mechanism is multifactorial, including factors relating to the environment and genetics, in addition to both disease-related and drug-related factors.^[45,46]^ Relatedly, the occurrence of at least 2 of these factors in combination may be of value in identifying those patients who are unlikely to be responsive to medical therapy.^[3,47–51]^ In our study, we proposed 2 explanations for genetic factors increasing DRE risk during infancy with epilepsy and NDD: First, during the neonatal or infancy period, early infantile epileptic encephalopathy accounts for a major part of genetic epilepsy, which are difficult to treat and often medically refractory.^[52]^ Second, on the basis of several hypotheses proposed on DRE, including hypotheses regarding pharmacokinetics, intrinsic severity, neural networks, gene variants, transporters, and targets,^[53]^ we assumed that a sustained “neuroimmunoinflammatory” status can be implicated as not only epileptogenic but also indicative of a drug-resistant profile.^[54]^ Additional relevant cell line– and animal model–based studies are thus warranted.

The present study had several limitations. First, while a risk of DRE in cases of genetic epilepsy was determined through this observational study, it is possible that the results may have been impacted by various confounding factors, such as the health status of the mother prior to and during pregnancy, socioeconomic status, pharmacological therapy of the children for conditions other than neurological conditions, poor nutrition (including malnutrition), or other environmental factors. Second, the size of the study sample was insufficient. We could not include patients of all types of genetic epilepsy in 1 study; moreover, 1 gene could have many different genotypes. Third, although our DRE definition was explicit, the seizure control results may have varied among physicians; that is, a DRE case for 1 physician could have attained seizure control with another. Fourth, although a comprehensive investigation was performed in all of the enrolled patients, an exact cause for epilepsy and NDD could not be determined in 19 patients; and thus the possibility of genetic factors’ involvement in those cases could not be completely dismissed, which was an inevitable confounder in the study. As such, further investigations of a thorough nature will be needed going forward in order to ascertain the related risks and pinpoint their underlying mechanisms in genetic and nongenetic epilepsy.

In summary, our study reveals that genetic factors act crucial role in younger children with epilepsy and NDD. Initiation of a genetic-based AED-development model, based on our current results, is warranted. In addition, the study can serve as a reference for further studies of epilepsy panel design and may also assist in the development of improved treatments and prevention strategies for DRE, particularly for drug control in more extensive and diverse genetic epilepsy, which has received insufficient attention thus far. That said, more data from relevant patients, as well as and more comprehensive studies of those data, will be needed in order to identify possible maternal, prenatal, perinatal, and postnatal confounders that could in turn help to clarify the effects of both genetic and nongenetic factors, as well as their associations with DRE.

## Acknowledgments

We wish to express our appreciation to the Genetic Medicine Laboratory of China Medical University Hospital, the Ministry of Science and Technology of Taiwan (MOST 107-2314-B-039-003), and the China Medical University Hospital Medical Research Department (#DMR-108–199 and # DMR-109–042) for giving assistance to this work.

## Author contributions

S-Y H collected the data, analyzed the data, and prepared the initial draft of the manuscript. C-H Lin took part in designing the study and wrote the final draft of the manuscript. The statistics for the study were compiled by I-C C, who also took part in the editing process and the revision of the tables. All of the authors read and approved of the final manuscript.

**Conceptualization:** Syuan-Yu Hong.

**Data curation:** Syuan-Yu Hong.

**Formal analysis:** I-Ching Chou, Syuan-Yu Hong.

**Investigation:** Syuan-Yu Hong.

**Methodology:** Syuan-Yu Hong.

**Resources:** Syuan-Yu Hong.

**Supervision:** Syuan-Yu Hong.

**Writing – original draft:** Chien-Heng Lin.

## Corrections

When originally published, Dr. Chien-Heng Lin appeared incorrectly as Chien-Hen Lin. This has now been corrected.
